# Micro 3D Printing Elastomeric IP-PDMS Using Two-Photon Polymerisation: A Comparative Analysis of Mechanical and Feature Resolution Properties

**DOI:** 10.3390/polym15081816

**Published:** 2023-04-07

**Authors:** Pieter F. J. van Altena, Angelo Accardo

**Affiliations:** Department of Precision and Microsystems Engineering, Faculty of Mechanical, Maritime and Materials Engineering (3mE), Delft University of Technology (TU Delft), Mekelweg 2, 2628 CD Delft, The Netherlands

**Keywords:** IP-PDMS, two-photon polymerisation, nanoindentation, Young’s modulus, elastomer

## Abstract

The mechanical properties of two-photon-polymerised (2PP) polymers are highly dependent on the employed printing parameters. In particular, the mechanical features of elastomeric polymers, such as IP-PDMS, are important for cell culture studies as they can influence cell mechanobiological responses. Herein, we employed optical-interferometer-based nanoindentation to characterise two-photon-polymerised structures manufactured with varying laser powers, scan speeds, slicing distances, and hatching distances. The minimum reported effective Young’s modulus (YM) was 350 kPa, while the maximum one was 17.8 MPa. In addition, we showed that, on average, immersion in water lowered the YM by 5.4%, a very important point as in the context of cell biology applications, the material must be employed within an aqueous environment. We also developed a printing strategy and performed a scanning electron microscopy morphological characterisation to find the smallest achievable feature size and the maximum length of a double-clamped freestanding beam. The maximum reported length of a printed beam was 70 µm with a minimum width of 1.46 ± 0.11 µm and a thickness of 4.49 ± 0.05 µm. The minimum beam width of 1.03 ± 0.02 µm was achieved for a beam length of 50 µm with a height of 3.00 ± 0.06 µm. In conclusion, the reported investigation of micron-scale two-photon-polymerized 3D IP-PDMS structures featuring tuneable mechanical properties paves the way for the use of this material in several cell biology applications, ranging from fundamental mechanobiology to in vitro disease modelling to tissue engineering.

## 1. Introduction

Two-photon polymerisation (2PP or TPP) has proven to be a versatile and powerful technology to fabricate true three-dimensional structures at the micro- and nanoscale with a resolution below the diffraction limit [1,2]. Two-photon polymerisation is a direct laser writing (DLW) technique that employs the non-linear absorption of two photons at the focal point of a femtosecond pulsed laser in a photosensitive material (also known as a photoresist). At the focal point, an ellipsoid-shaped volume of the resist, known as a voxel, is polymerised if the absorption energy exceeds the activation energy of the photoinitiator molecules [3,4]. Complex geometries can be created via 2PP by scanning the voxel through the resist, often without needing additional supporting structures. Further details on the 2PP printing process can be found in previous research performed by Farsari and Chichkov [5], Ovsianikov and Chichkov [6], Malinauskas et al. [7], and Bunea et al. [8]. Various resists, based on different polymers and photoinitiators [9], are available commercially in different configurations: gels, (viscous) liquids, or amorphous solids [3,10]. These materials include hard polyacrylic resins (e.g., IP-Dip), soft hydrogel acrylic esters (e.g., GelMA), epoxides (e.g., SU8), and organic/inorganic hybrid materials (e.g., sol-gels) [11,12,13]. The Young’s moduli of such polymers range from a few kPa for hydrogel-based materials, such as GelMA and hyaluronic acid [14], to hundreds or thousands of kPa for poly(ethylene glycol) diacrylate (PEGDA)-based hydrogels [15], and up to several GPa for materials such as SU-8, Ormocomp, and IP-Dip [16]. However, the Young’s modulus of each material can be tuned within specific ranges by altering the fabrication parameters, which influences the cross-link density [16]. In their work, Lemma et al. [16] proposed a quasi-linear relationship between the laser power and the resulting GPa range of 2PP materials’ Young’s moduli, while in a follow-up study, they obtained a stiffness gradient by altering the laser power throughout a print [4]. The ability to tune the Young’s modulus, in this case, was limited to 2PP polymers featuring GPa Young’s moduli, which have already been extensively used for cell mechanobiology [17,18,19] and in vitro disease modelling applications [20,21]. The main focus of the present work is, on the other hand, to focus on the tuneable mechanical properties and the achievable feature resolution of a soft (kPa-MPa YM) elastomeric material, IP-PDMS, which could find application in the context of neuronal engineered microenvironments [22] as the brain is one of the softest tissues in the body. 

IP-PDMS (Nanoscribe GmbH & Co. KG, Karlsruhe, Germany) is a photocurable type of polydimethylsiloxane (PDMS). This proprietary photoresist has a nominal Young’s modulus of 15.3 MPa according to the producer’s specifications [8]. IP-PDMS is, thus, approximately 100 times softer compared with other proprietary Nanoscribe materials [8]. Microscale architectures made from soft elastomeric (bio)materials like hydrogels and PDMS, are extremely interesting for biomedical applications, such as cell scaffolding and mechanobiological studies, as cells can deform the material, thus not generating large tension forces and retaining their shapes [8,22]. In addition, soft polymeric materials can also be employed in other fields, such as microfluidics, sensors, and actuators. 

However, all these applications require a thorough mechanical and morphological characterisation to obtain robust structural designs as well as reproducible and reliable results, thus allowing for better interpretation of experimental observations [16]. Predicting the mechanical properties of 2PP-printed materials is challenging as they depend on various parameters. Previous works have investigated the tunability of conventional PDMS by using different mixing ratios of the base (pre-polymer) and the hardener (curing agent), temperatures, and curing times [23,24,25]. Herein, we focus instead on characterising the mechanical properties of the novel 2PP IP-PDMS elastomeric material with respect to the 2PP printing parameters: laser power, scan speed, slicing distance, and hatching distance. By employing a nanoindenter equipped with a spherical probe, which is particularly suitable for the characterisation of soft materials [26], we measured the effective Young’s modulus (YM) of IP-PDMS in the presence of different sets of printing parameters. The results indicate a high dependence of the effective YMs of the 2PP-printed constructs on the printing parameters, with effective Young’s moduli ranging from 350 kPa to 17.8 MPa. The results also indicate that the effective YM of IP-PDMS constructs is lowered by an average of 5.4% in an aqueous environment compared with measurements in air, which must be taken into account for cell biology experiments. In addition, we developed a printing strategy to obtain micrometric-thin beam structures with large overhangs. The reported results can thus serve as a basis for not only the future development of 3D cell scaffold applications but also biosensor and microfluidics ones employing this elastomeric material. 

## 2. Materials and Methods

### 2.1. Materials

IP-PDMS, a proprietary photosensitive acrylate elastomeric polymer (Nanoscribe GmbH & Co. KG, Karlsruhe, Germany), was employed for the fabrication of all the two-photon-polymerized structures. IP-PDMS is, according to the manufacturer, a non-cytotoxic hydrophobic material according to ISO10993-5. Indium tin oxide (ITO)-coated soda–lime substrates were purchased as well from Nanoscribe GmbH. All other chemicals (acetone, isopropanol, ethanol, MAPTMS, and Novec7100) were purchased from Sigma-Aldrich, Germany.

### 2.2. Two-Photon Polymerization Fabrication Protocol

For the fabrication of the 2PP structures, a commercial two-photon direct laser writing setup was employed (Photonic Professional GT+ system, Nanoscribe GmbH & Co. KG, Karlsruhe, Germany). First, the .stl designs of the structures were developed using the CAD software Solidworks 2021 (Dassault Systems SolidWorks Corp., Vélizy-Villacoublay, France). The .stl files were then imported into the proprietary DeScribe software (Nanoscribe GmbH & Co. KG, Karlsruhe, Germany), a slicing software that allows programming voxel trajectories, setting the printing parameters and exporting GWL print files. The software enables the slicing (dividing the geometry into vertically stacked planes in the z direction) and hatching (dividing the sliced planes into voxel trajectory lines in the xy plane) of 3D .stl designs. The printing parameters that were tuned in the current study were slicing distance, hatching distance, laser power, and scan speed. The system employs a femtosecond fibre laser source with a wavelength of 780 nm, a standard maximum laser power of 50 mW (corresponding to 100% laser power), a repetition rate of 80 MHz, and a pulse length of 100 fs. To focus the laser light on the samples, a LCI Plan-Neofluar 25×/0.8 Imm Corr DIC M27 (Carl Zeiss, Oberkochen, Germany) microscope objective was employed. The objective was operated in dip-in laser lithography (DiLL) mode during the fabrication (i.e., with the lens dipped directly in the IP-PDMS photopolymer). 

All structures were printed on 25 × 25 × 0.7 mm^3^ (l × w × h) indium tin oxide (ITO)-coated soda–lime substrates. Before printing, the substrates were first cleaned with acetone and isopropanol using a lint-free wipe. The samples were then further cleaned and activated using an oxygen plasma setup (Diener electronic GmbH + Co. KG) for 10 minutes at a power of 80 W and an oxygen gas flow rate of 5 cm^3^/min. To improve the adhesion of the IP-PDMS structures to the substrate, a silanisation protocol, consisting of a 2-hour immersion of the substrate in a 2% *v/v* 3-(trimethoxysilyl)propyl methacrylate (MAPTMS)/ethanol (99.8%) solution, was performed. A drop of IP-PDMS was cast on top of the silanised samples, after which the printing procedure was started. After the 2PP process, all samples were chemically developed in isopropanol for 10 minutes to remove the unexposed IP-PDMS, after which a 2nd rinse of 1 minute in clean isopropanol was used to remove any remaining unpolymerised material. The samples used for morphological characterisation were then immersed in Novec 7100 engineered fluid to prevent their collapse due to surface tension. All samples were left to air dry under a fume hood. 

### 2.3. Fabrication of Specimens for Mechanical and Morphological Characterisation

To measure the influences of the printing parameters on the effective YM of IP-PDMS, circular pedestals with a diameter of 350 µm and a height of 50 µm were printed. This size of the pedestal allows the simple optical alignment of the indentation probe over a specimen and obtaining a measurement of the material’s YM without sensing the stiffer soda–lime underlying substrate. In addition, the large diameter prevents any effects of the edges of the pedestals from interfering with the measurements. The pedestals were printed in large arrays with 3 × 3 subarrays for each variation of the printing parameters (thus, n = 9 per parameter). Printed markers indicate the separation between specific subsets, simplifying identification during the nanoindentation experiments (see Supplementary Figure S1 in the Supplementary Material). The printing parameters employed for the IP-PDMS pedestals were scan speeds ranging from 50 mm/s to 100 mm/s with steps of 10 mm/s; laser powers ranging from 25 mW to 45 mW with steps of 5 mW; and slicing and hatching both kept at 0.3 µm. Concerning the experiments with varying slicing distances, hatching distances, and environmental conditions, we employed 2 laser powers (35 mW and 40 mW) and 2 scan speeds (60 mm/s and 80 mm/s). The overall parameter set is reported in Table S1 in the Supplementary Material). Prints with parameters outside the mentioned ranges either did not lead to consistent results, had severe micro explosions due to bubble formation, or were delaminated during chemical development. 

Since pedestals have elementary geometries, additional designs were developed to assess the limits of the high-resolution printing of large overhangs featuring micrometric IP-PDMS features. To achieve thin and narrow beams with large overhangs, we programmed the voxel trajectory in the DeScribe software (Figures S2 and S3 in the Supplementary Material). The beams were printed with a single slice (one voxel height) by scanning the voxel in the transverse direction (perpendicular to the longitudinal axis of the laser beam) with varying beam widths, laser powers, scan speeds, and hatching distances. The thickness of a beam depends on the voxel height, while the user can set the width. Before printing the beams, 2 pedestals with dimensions of 20 × 30 × 20 µm^3^ (l × w × h) were printed at 3 different distances (30 µm, 50 µm, and 70 µm), with slicing and hatching of 0.3 µm, a laser power of 40 mW, and a scan speed of 90 mm/s. The beams were printed with hatching distances of 0.2, 0.25, and 0.3 µm, scan speeds ranging from 3 mm/s to 5 mm/s with a 0.5 mm/s step, and laser powers of 42.5 mW, 45mW, and 47.5 mW (Table S2 in the Supplementary Material). 

### 2.4. Mechanical Nanoindentation

The Piuma nanoindenter (Optics11 Life b.v., Amsterdam, The Netherlands) was employed to measure the mechanical properties of the printed pedestals. A probe with a cantilever stiffness of 42.7 N/m and a tip radius of 24.5 µm was used for all indentations. A spherical tip is commonly used for indenting soft polymers as it avoids damage to the substrate, minimises the plastic deformation of the sample, and prevents stress concentrations [27]. In order to avoid artefacts in the measurement, the maximum indentation depth recommended by the manufacturer is 16% of the radius of the tip, thus, 3.92 µm, and should not exceed 5–10% of the sample thickness, as this could introduce substrate effects. For an indentation of 1 µm, it is possible to measure Young’s moduli in the range of 48.5 kPa to 121 MPa with the employed probe. The probe was calibrated on a glass substrate before each experiment and after switching to another medium to linearize the interferometric signal and determine the probe calibration factor (i.e., the factorial difference between the cantilever bending at the signal point and the cantilever bending at the contact point). The spherical tip was then aligned with the centre of an IP-PDMS pedestal using the bottom view camera of the nanoindenter, and the indentation sequence was started. Nine indentations were performed on different IP-PDMS pedestals for each printing parameter. The effective YMs were obtained automatically from the load/indentation curves, using the Hertzian contact fit. The Hertzian contact model derives a material’s effective YM from the loading region of the indentation curve and is the most suitable model for soft (bio)materials [15,28]. This method employs the load (*P*), the radius of the tip (*R*), and the indentation depth (*h*) to calculate the effective Young’s modulus (*E_eff_*) via Equation (1) [28].

(1) $$P=\frac{4}{3}E_{eff}R^{1/2}h^{3/2}$$

The data were imported into Matlab (MathWorks, Inc., Natick, MA, USA) to plot them for each printing parameter. One set of experiments was performed on the IP-PDMS pedestals immersed in deionised water to assess the effect of an aqueous environment on the 2PP IP-PDMS structures. The samples were immersed in water at room temperature for two hours before starting the experiment.

### 2.5. Scanning Electron Microscopy

Scanning electron microscope (SEM) images were acquired using a JSM 6010LA (JEOL Ltd., Tokyo, Japan) setup with an acceleration voltage of 6 to 20 kV at 0 and 65-degree tilt angles in the secondary electron imaging mode (SEI). Before SEM imaging, the samples were coated with a thin layer of gold using the JFC-1300 Auto Fine (JEOL Ltd., Tokyo, Japan) low-voltage planar magnetron sputter coater with argon gas. The samples were sputtered twice at 30 mA for 30 seconds at a distance of 25 mm to the target, once from the top and once at a 45-degree tilt, resulting in an approximate layer thickness of 10 nm. The top surfaces of the ITO-coated substrates were connected to the SEM holder with carbon tape to ensure good electrical conductivity, thus preventing charging. 

## 3. Results and Discussion

### 3.1. Mechanical Characterisation of Two-Photon-Polymerised IP-PDMS Microstructures

**Figure 1:**
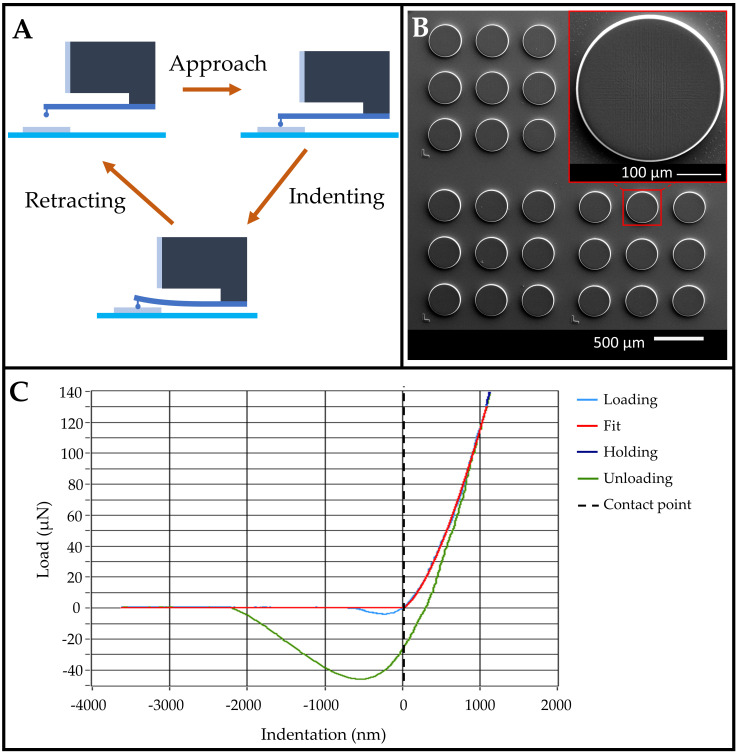
Indentation experiments. (**A**) Schematic indentation procedure with an interferometer-based nanoindenter (Piuma, Optics 11). (**B**) SEM micrograph of three sets of pedestal arrays employed for the indentation experiment. Each set consists of nine replicates, with the inset showing a detailed view of one pedestal. (**C**) Typical load/indentation plot with Hertzian fit. The contact point is located at an indentation depth of zero. Before the contact point, a snap-in occurs, and probe/IP-PDMS adhesion is visible in the unloading curve.

Figure 1A illustrates the indentation process with the approach, indenting, and retracting steps highlighted. These steps were automatically performed on each of the pedestals after the manual alignment of the probe over the IP-PDMS pedestals. Figure 1B shows three sets of printed IP-PDMS pedestals, each consisting of nine replicates. The different sets are easily identifiable by the markers and inter-sets spacing. The inset in Figure 1B shows a close-up SEM micrograph of the top surface of a pedestal. These pedestals were used for mechanical characterisation before SEM imaging. 

The roughness of a pedestal is caused by shrinkage after printing and during chemical development. Figure 1C shows a representative load/indentation curve of a nanoindentation experiment where, first, the substrate is approached until the probe makes contact with it (contact point at 0 nm). A clear snap-in occurs right before the contact point (light blue line). From the contact point, the indentation takes place, still during the loading procedure. The maximum indentation in this figure is ≈ 1.2 µm, well below the 3.92 µm limit for the employed probe (see Section 2.4). The resulting load/indentation curve is employed to determine the effective Young’s modulus by employing the Hertzian contact model (red line). Before unloading, a short pause (1 s) takes place (holding line) to ensure the probe is settled before retraction. The unloading follows a different curve as adhesion of the probe to the sample occurs (green line).

**Figure 2:**
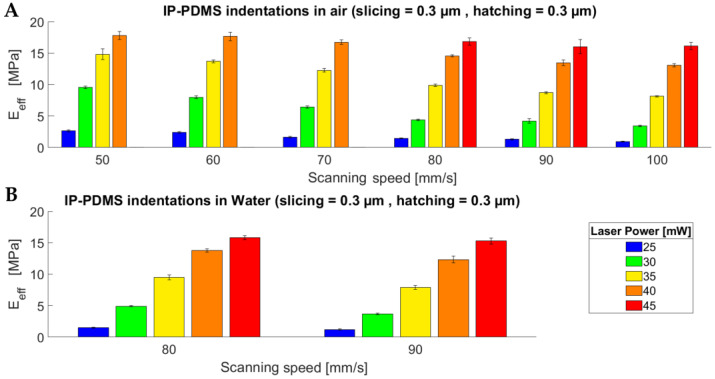
Indentation results showing the influence of the scanning speed on the effective Young’s modulus of IP-PDMS. (**A**) Indentations in air. (**B**) Indentations in water (room temperature).

**Figure 3:**
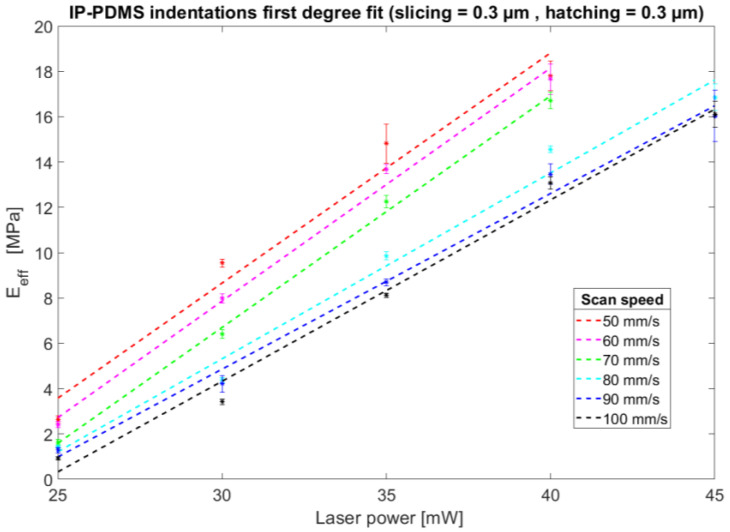
First-degree polynomial fit showing the linear relationship between IP-PDMS’s Young’s modulus and employed laser power at different scan speeds.

Figure 2 shows the results of the indentation experiments performed with the IP-PDMS microstructures either in an air environment or dipped in water. From Figure 2A, it is clear that an increase in scan speed, with a constant slicing and hatching distance of 0.3 µm, results in a lower effective YM. This can be explained by the lower dose, thus resulting in a lower degree of polymerisation. The measurement of the pedestals with a 45 mW laser power is not present for the lower scan speeds as microexplosions during printing caused bubble formation due to local heating of the photopolymer, thus resulting in unreliable results. The minimum measured effective YM, with a constant slicing and hatching distance of 0.3 µm, was 900 kPa with a laser power of 25 mW and a 100 mm/s scan speed, while the maximum one was 17.8 MPa with a laser power of 40 mW and a scan speed of 50 mm/s (and a slicing and hatching distance of 0.3 µm in air conditions).The exact effective EY values are reported in Table S3. Figure 3 shows the linear relationship between the IP-PDMS effective YM and employed laser power at different scan speeds. Interestingly, a change in the slopes of the curves seems to occur between 70 mm/s and 80 mm/s. Similar linear behaviour can be found for the relationship between the Young’s modulus and employed scanning speed (Figure S4).

Figure 2B shows the effect of the immersion in water of the IP-PDMS pedestals, two-photon-polymerised at 80 and 90 mm/s, and different laser powers. Our results indicate that room temperature water lowers the measured effective Young’s modulus by an average of 5.4%. The minimum effective YM reported for the set of indentations performed in water was 1.2 MPa with a constant slicing and hatching distance of 0.3 µm, laser power of 25 mW, and with a 90 mm/s scanning speed (while the measurement for the same sample in air was 1.3 MPa). In summary, the influence of water on IP-PDMS cannot be neglected and should be taken into account, especially for cell culture applications where even slight variations in the Young’s modulus can have a significant impact on cell fate.

**Figure 4:**
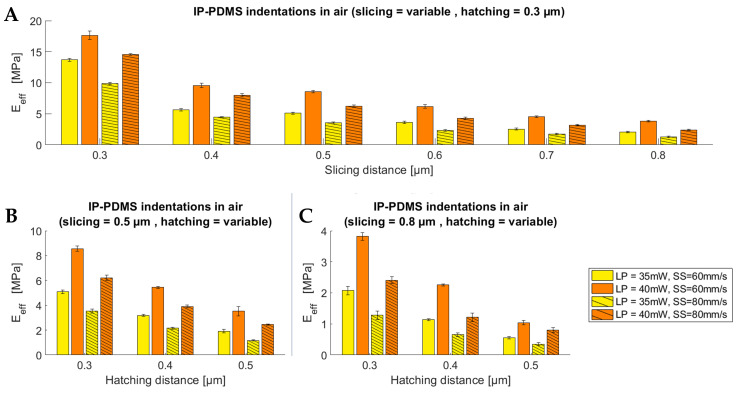
Indentation results showing the influence of slicing and hatching distance on the effective Young’s modulus of IP-PDMS. (**A**) Influence of the slicing distance on the effective Young’s modulus with a constant hatching distance of 0.3 µm. (**B**) Influence of the hatching distance on the effective Young’s modulus with a constant slicing distance of 0.5 µm. (**C**) Influence of the hatching distance on the effective Young’s modulus with a constant slicing distance of 0.8 µm.

Figure 4A–C shows the significant effect of the slicing and hatching distances on the effective Young’s modulus. Figure 4A shows that a maximum effective Young’s modulus (YM) of 17.7 MPa was found for a slicing distance of 0.3 µm, while the lowest YM was 2.4 MPa for a slicing distance of 0.8 µm with a constant hatching distance of 0.3 µm. Figure 4B,C depicts the effect of different hatching distances on the effective YM, where a decreasing YM was reported for an increasing hatching distance. Figure 4A shows that an increase in the slicing distance from 0.3 µm to 0.4 µm results in a decrease in the YM of 46% (17.7 MPa to 9.6 MPa, respectively) with a laser power of 40 mW and a scanning speed of 60 mm/s. Similarly, Figure 4B shows that increasing the hatching distance from 0.3 µm to 0.4 µm can result in a decrease in the YM of over 37% (from 8.6 MPa to 5.4 MPa, with the same laser power and scan speed as mentioned above). Figure 4C shows that a minimum YM of 350 kPa can be obtained by employing a slicing distance of 0.8 µm, a hatching distance of 0.5 µm, a laser power of 35 mW, and a scanning speed of 80 mm/s. 

The indentation results thus show that the effective YM is highly dependent on the fabrication parameters, ranging from approximately 350 kPa to 17.8 MPa. As expected, a lower dose (by either lowering the laser power, increasing the scan speed, or increasing the slicing and hatching distances) results in a lower effective YM, where the effect of the laser power is more pronounced. Increasing the photon flux density, which is proportional to the laser power and inversely proportional to the scanning speed [13], increases the degree of conversion of the polymer, which leads to an increase in the Young’s modulus [29]. Similarly, lower hatching and slicing distances result in higher overall effective YM values as the dose per volume is higher. This is caused by the overlap of voxel lines, which is larger for smaller hatching and slicing distances, causing the same volume to be exposed multiple times, thus increasing the dose. Therefore, changing the printing parameters leads to a change in the degree of conversion, which is directly coupled to the Young’s modulus of the printed structure.

By varying these properties throughout a print, stiffness gradients can be obtained. These gradients can be exploited to study the effect of mechanotransduction on cells, as nearly all phenomena in cell biology are affected by substrate stiffness to some degree [30]. Compared to conventional PDMS, the reported range of achievable Young’s moduli of IP-PDMS is significantly larger. For conventional PDMS, Wang et al. [31] reported YMs ranging from 570 kPa to 3.7 MPa. Furthermore, for applications in which the Young’s modulus is important, such as cell microenvironments and deformable structures, it is essential to measure it in aqueous environments to unveil possible correlations (e.g., for studies about the relationship between a material’s Young’s modulus and cell differentiation [22,32,33]). The effect of swelling on the Young’s modulus is indeed crucial for biological applications, as, in such circumstances, the IP-PDMS is in contact with liquids. Kappert et al. [34] found a swelling degree of 2.3% for PDMS in water. The latter should be investigated at incubator temperatures for photocurable IP-PDMS before performing biological experiments as it could influence the results. Interestingly, papers focusing on the effect of the Young’s moduli of polymers on cellular behaviour often neglect this effect.

### 3.2. Morphological Characterisation of Two-Photon-Polymerised IP-PDMS

**Figure 5:**
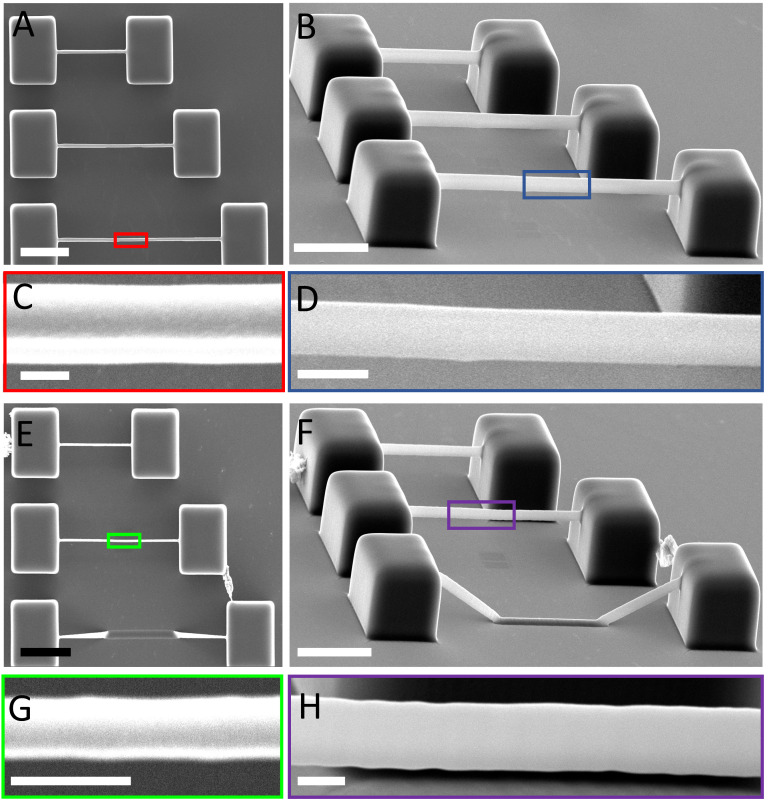
Representative SEM micrographs of 2PP-printed IP-PDMS beams. (**A**) Top view of beams with nominal lengths of 30, 50, and 70 µm and a nominal width of 1 µm (hatching distance of 0.25 µm, laser power of 45 mW, and scan speed of 3 mm/s; scale bar = 20 µm). (**B**) Tilt view of 65 degrees of the beams in A (scale bar = 20 µm). (**C**) Top view close-up of the beam with a length of 70 µm. The beams printed with these settings have an average width of 1.46 ± 0.11 µm (n = 3; scale bar = 1 µm). (**D**) Sixty-five-degree tilt view of the side of the printed beam indicated in B. The measured thickness of the beam is 4.49 ± 0.05 µm (n = 3; scale bar = 5 µm). (**E**) Top view of beams with nominal lengths of 30, 50, and 70 µm and a nominal width of 1 µm (hatching distance of 0.3 µm, laser power of 42.5 mW, and scan speed of 4.5 mm/s; scale bar = 20 µm). Note the 70 µm beam collapsed. (**F**) Tilt view of 65 degrees of the same beams as in E (scale bar = 20 µm). (**G**) Top view close-up of the beam with a length of 50 µm. The beams printed with these settings have an average width of 1.03 ± 0.02 µm (n = 3; scale bar = 2 µm). (**H**) Sixty-five-degree angle view of the side of the printed beam indicated in F. The measured thickness of the beam is 3.00 ± 0.06 µm (n = 3; scale bar = 2 µm).

To employ 2PP-printed microstructures for cell biology experiments, it is important to mimic the features of the in vivo cellular environment. This is not limited to the mechanical properties but also involves obtaining feature sizes in the same range as that of the geometric features of cells and their extracellular matrix. Features in the same size range of cells allow fostering the interaction between a (bio)material and a cell and enable the visualisation of 3D cell networks without having cell-overlap issues that might hamper the detection of immunofluorescence markers [21]. However, obtaining small features with overhangs in the presence of soft 2PP materials remains a challenge. The reported morphological characterisation of 2PP-printed IP-PDMS microstructures shows that very thin and narrow beams with long unsupported overhangs can be printed with IP-PDMS using our printing strategy. We printed arrays of double-clamped beams (fixed at both ends) with a designed width of 1 to 4 µm, a length of 30, 50 and 70 µm, and a thickness of a single slice (see Section 2.3 for further details). First, two supporting pedestals were printed, after which each beam was printed starting from the first pedestal until the beam was eventually connected to the second pedestal. Figure 5A,B shows the resulting beams with nominal lengths of 30, 50, and 70 µm. Beams longer than 70 µm did not lead to reproducible results. The maximum length of a printed beam was 70 µm with a minimum width of 1.46 ± 0.11 µm (n = 3) and a beam thickness of 4.49 ± 0.05 µm (n = 3), as depicted in Figure 5A–D. The minimum beam width of 1.03 ± 0.02 µm (n = 3) was achieved for a beam length of 50 µm, with a beam thickness of 3.00 ± 0.06 µm (n = 3), as shown in Figure 5E–H.

By printing single slices with hatch lines perpendicular to the longitudinal axes of the double-clamped beams (Figure S3), we obtained narrow and thin beams with overhangs of up to 70 µm. However, this approach only yielded successful results with very high laser powers (40–50 mW) and low scan speeds (3 to 5 mm/s). Furthermore, IP-PDMS features a relatively low viscosity (~100 mPa·s at 25 °C, according to the manufacturer), which causes it to spread over the substrate, making it challenging to print on multiple substrates in series. This also provokes the time-dependent deformation of weak printed first slices before and during the printing of a second slice on top of the previous one (Figure S5 and Video S1).

In addition, when thin and long overhanging hatch lines are printed, the second hatch line tends to deform the first printed line (which caused the collapse of the 70 µm long beam in Figure 5F). In comparison, IP-S, another proprietary 2PP Nanoscribe material featuring a YM in the GPa range, has a viscosity of 13,600 mPa·s at 20 °C. To overcome the issues of peeling and bending of the first printed layers, we, therefore, developed a printing strategy consisting of two-photon polymerising a single slice using direct writing commands to program the voxel trajectory, resulting in straight beams with small widths and small heights (for further details, see Section 2.3 and Figure S3). However, the voxel size caused the beams to have relatively large dimensions along the Z direction for all prints. According to the manufacturer, the voxel’s diameter is approximately 0.6 µm with an aspect ratio of 6, meaning the height of the voxel is approximately 3.6 µm, which is similar to the minimum Z height (i.e., thickness) of the beams. 

**Figure 6:**
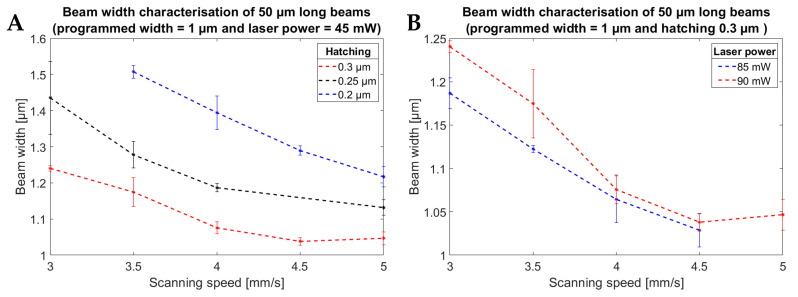
Plots of the measured widths of 50 µm long beams. The designed width of the beams was 1 µm. (**A**) Graph showing the effect of both the scanning speed and the hatching distance on the resulting beam widths (constant laser power at 45 mW). (**B**) Effect of the laser power on the widths of the beams at different scanning speeds (constant hatching distance of 0.3 µm).

Finally, Figure 6 shows the effects of the hatching distance (Figure 6A) and the laser power (Figure 6B) on the resulting widths of the beams. Figure 6A shows that a larger hatching distance and higher scanning speed, and thus a lower dose, results in narrower beams. As expected, Figure 6B shows that a lower dose (lower laser power) results in narrower beams compared with a higher laser power.

## 4. Conclusions

The reported investigation shows the tunability of the soft elastomeric polymer IP-PDMS by employing 2PP. The Young’s modulus could be tuned, depending on the manufacturing parameters, in the range of 350 kPa to 17.8 MPa (measured via nanoindentation). The results provide an initial selection guide for choosing the appropriate printing parameters with respect to the required mechanical properties. In particular, our study shows that the Young’s modulus of IP-PDMS can be tuned to be two to three orders of magnitude smaller than that of commercial 2PP acrylate polymers while still being able to manufacture freestanding 3D structures with dimensions close to the one of mammalian cells. To the best of our knowledge, this is the first comparative mechanical and morphological study performed on IP-PDMS. The tuneable Young’s modulus can be exploited for mechanobiological studies to investigate the effects of 2D, 2.5D, and 3D structures’ stiffness on the mechanotransduction pathways of cells belonging to soft tissues such as the brain. Similar characterisation protocols can also be employed for other soft 2PP materials, such as PEGDA and GelMA [15,35]. Future research should investigate the effects of other factors that can influence mechanical properties of IP-PDMS, such as UV sterilisation and incubation temperatures. In light of these results, soft photosensitive elastomeric materials provide new opportunities for 3D cell scaffolding experiments, as their mechanical properties can be tuned on demand to create structures featuring stiffness gradients.

## Data Availability

The data that support the findings of this study are available from the corresponding author upon reasonable request.

## Supplementary Materials

The following supporting information can be downloaded at https://www.mdpi.com/article/10.3390/polym15081816/s1.
