# Supplementary material for: Micro 3D Printing Elastomeric IP-PDMS Using Two-Photon Polymerisation: A Comparative Analysis of Mechanical and Feature Resolution Properties
Source: Polymers (Basel). 2023 Apr 7;15(8):1816. doi: 10.3390/polym15081816 (PMC10144803; doi:10.3390/polym15081816)
Supplement: Supplementary file 1 [file polymers-15-01816-s001.zip › Supplementary Material.pdf]

Supplementary information

Micro 3D Printing Elastomeric IP-PDMS Using Two-Photon Polymerisation: A Comparative Analysis of Mechanical and Feature Resolution Properties

Pieter F. J. van Altena and Angelo Accardo *

Department of Precision and Microsystems Engineering, Faculty of Mechanical, Maritime and Materials Engineering (3mE), Delft University of Technology (TU Delft), Mekelweg 2, 2628 CD Delft, The Netherlands
*Correspondence: a.accardo@tudelft.nl; Tel.: +31-15-27-81610

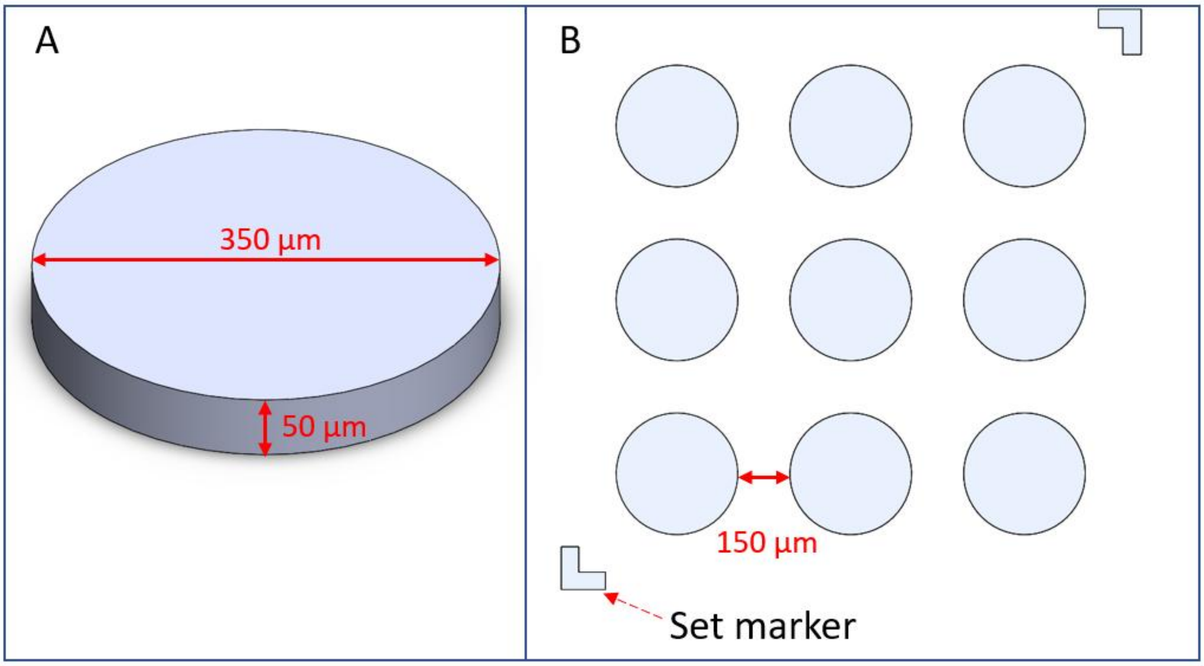

Figure S1. (**A**) Pedestal design with a diameter of 350 µm and a height of 50 µm. One pedestal fits inside the printfield of the 25x objective. (**B**) Set of replicate pedestals printed with the same parameters. The set markers are visual aids during the experiment. The pedestals and markers are all printed sequentially.

Table S1: The ranges of printing parameters employed for printing the IP-PDMS pedestals used for the indentation experiments.

Slicing (range) [µm]	Hatching (range) [µm]	Laser power range [mW]	Scan speed range [mm/s]
0.3	0.3	25 to 45	50 to 100
0.3 to 0.8	0.3	35 and 45	60 and 80
0.5	0.3 to 0.5	35 and 45	60 and 80
0.8	0.3 to 0.5	35 and 45	60 and 80

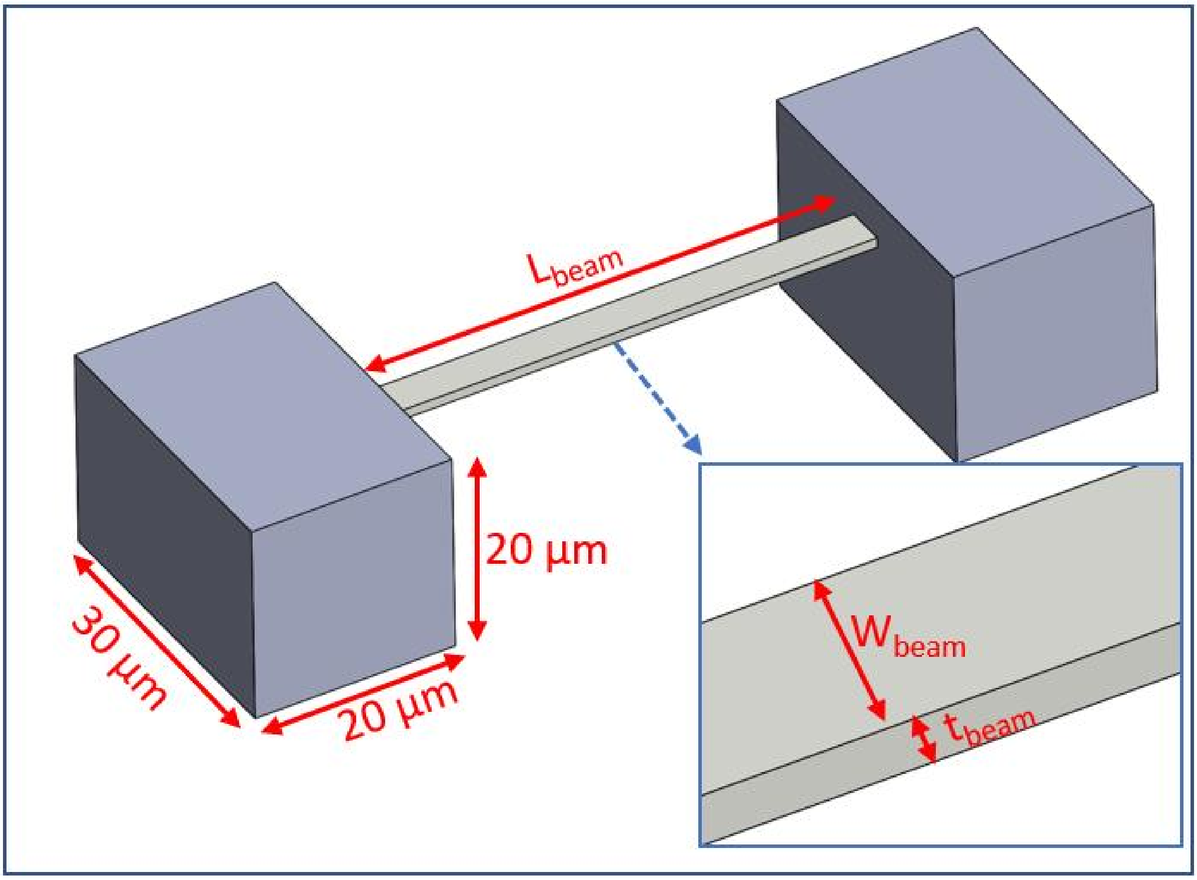

Figure S2: Design of the double-clamped beam. The pedestals of the beam (dark grey) are printed from a .STL file while the beam (light grey) is printed by programming the voxel trajectory through GWL code. The length of the beam (L_{beam}) and the width of the beam (W_{beam}) were varied, while the thickness of the beam (t_{beam}) was designed to be a single slice (voxel height). The beam is printed at the height of 15 µm from the substrate.

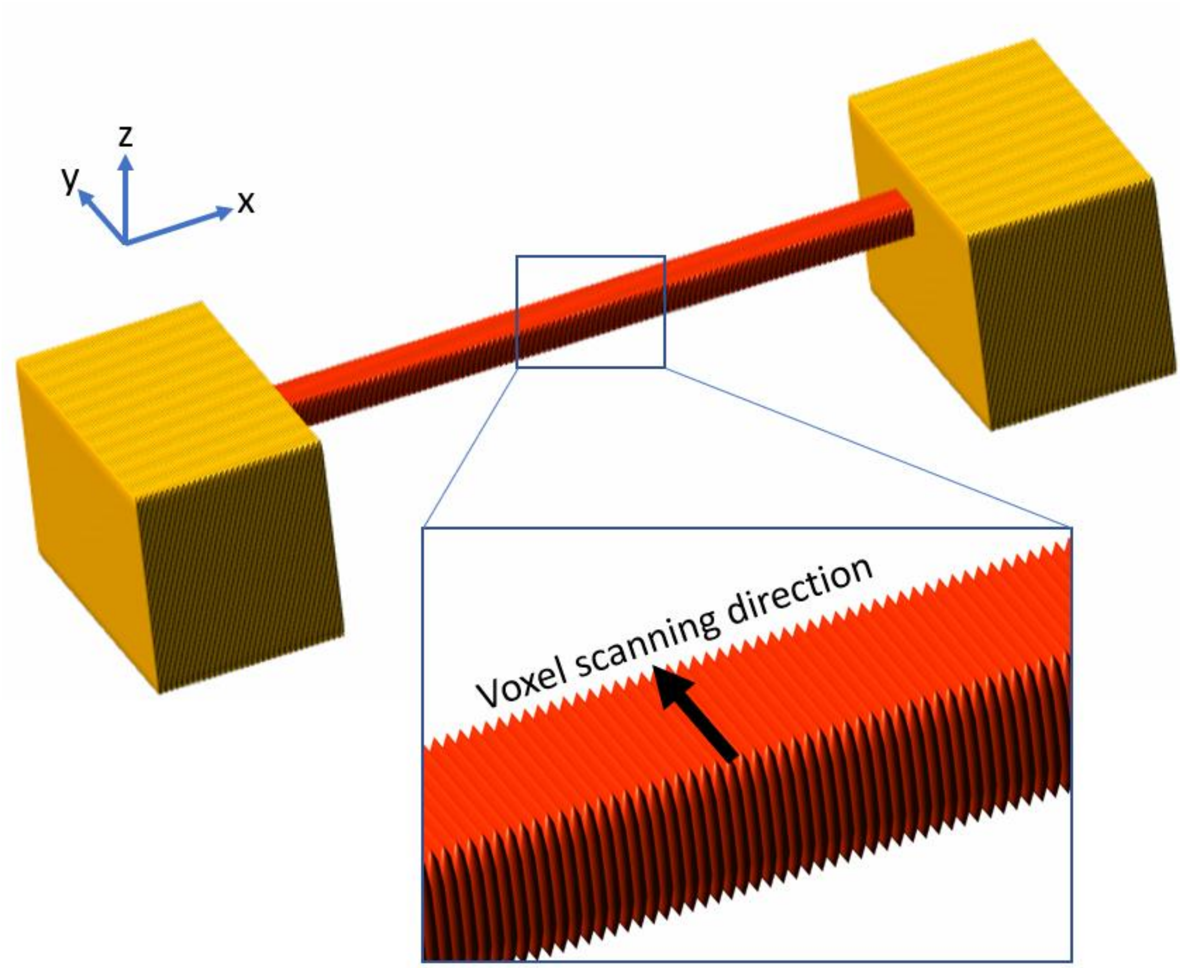

Figure S3: Beam printing strategy. The laser voxel scans a line in the positive y direction (traverse direction of the beam), then moves to the next line (in positive x direction, longitudinal axis) and scans that line again in positive y direction.

Table S2: Table with the (range of) printing parameters for the printing of the array of beams

	**Slicing (range) [µm]**	**Hatching (range) [µm]**	**Laser power range [mW]**	**Scan speed range [mm/s]**	**Beam width (range) [µm]**
**Beam**	n.a.	0.2–0.3 (step 0.05)	42.5 to 47.5 (step 2.5)	3 to 5 (step 0.5)	1 to 4 (step 0.5)
**Pedestals**	0.3	0.3	40	90	n.a.

Table S3: Indentation results (n=9) accompanying figure 2A of main text.

	Scanning speed [mm/s]											
	**50**		**60**		**70**		**80**		**90**		**100**	
**LP**	YM [Mpa]	stdev [MPa]	YM [Mpa]	stdev [MPa]	YM [Mpa]	stdev [MPa]	YM [Mpa]	stdev [MPa]	YM [Mpa]	stdev [MPa]	YM [Mpa]	stdev [MPa]
**25**	2.64	0.17	2.41	0.14	1.64	0.12	1.44	0.06	1.29	0.11	0.93	0.07
**30**	9.54	0.18	7.98	0.20	6.41	0.20	4.38	0.12	4.21	0.37	3.42	0.12
**35**	14.81	0.85	13.68	0.21	12.25	0.28	9.86	0.19	8.69	0.16	8.12	0.10
**40**	17.80	0.66	17.65	0.68	16.72	0.36	14.56	0.16	13.47	0.44	13.06	0.27
**45**	n.a.	n.a.	n.a.	n.a.	n.a.	n.a.	16.86	0.58	16.03	1.13	16.11	0.57

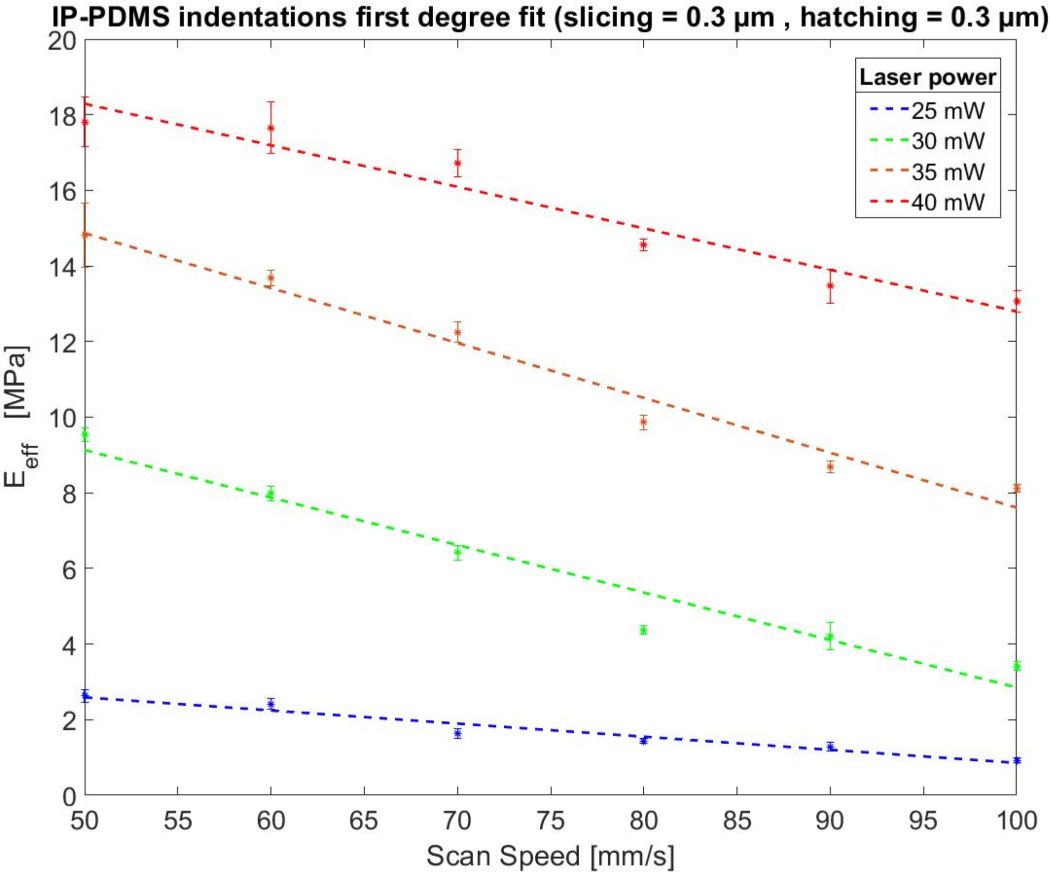

Figure S4: Linear relationship bewettn IP-PDMS Young’s modulus and employed scanning speed.

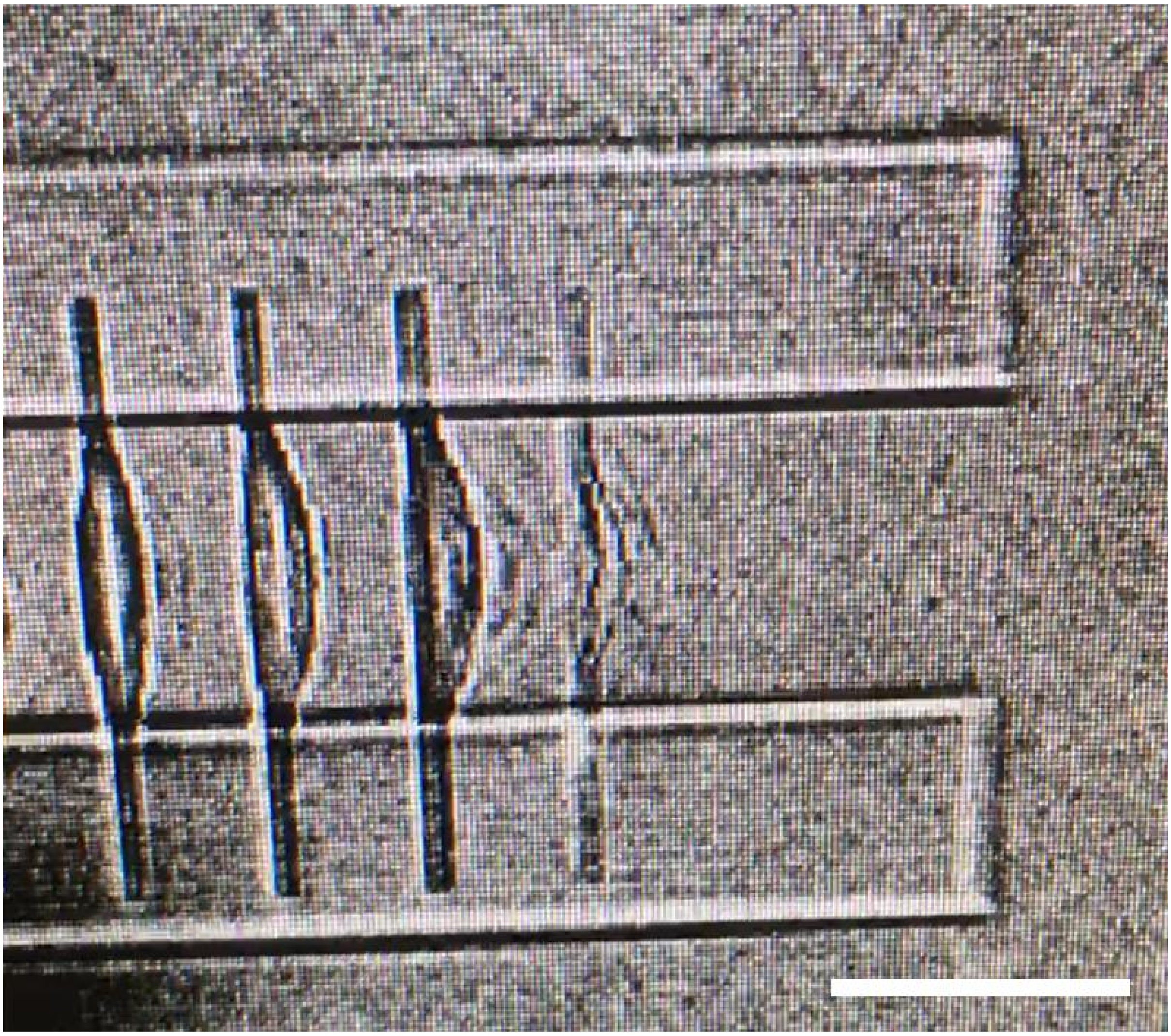

Figure S5: Delamination occuring when printing IP-PDMS beams with hatch lines in the longitudinal direction. Scale bar is 30 μm. See supplementary video for additional details.
